# Cigarette smoking and associated factors among in-school adolescents in Jamaica: comparison of the Global Youth Tobacco Surveys 2000 and 2006

**DOI:** 10.1186/1756-0500-1-55

**Published:** 2008-07-28

**Authors:** Adamson S Muula, Seter Siziya, Emmanuel Rudatsikira

**Affiliations:** 1 Department of Community Health, University of Malawi, College of Medicine, Blantyre, Malawi; 2 Department of Community Medicine, University of Zambia, School of Medicine, Lusaka, Zambia; 3 Department of Global Health, Loma Linda University, School of Public Health, Loma Linda, California, USA; 4 Department of Epidemiology and Biostatistics, Loma Linda University, School of Public Health, Loma Linda, California, USA

## Abstract

**Background:**

We conducted this study to estimate the correlates of current cigarette smoking among in-school adolescents in Jamaica 2006 and compare prevalence of smoking and associated factors between 2000 and 2006.

**Results:**

In 2006, 1854 participated of whom 49.5 were males and 50.5% females. 1752 adolescents, 48.8% male and 51.2% females participated in the 2000 survey. Between 2000 and 2006, the prevalence of smoking among Jamaican school-going adolescents went up slightly from 15.2% to 16.7% but this was not statistically significant (p = 0.22). The perception that smoking is not harmful increased from 10.9% to 15.9% while parental smoking decreased from 39.4% to 35.5%. There was a decrease in the rates of adolescents exposed to tobacco adverts on billboards (p-value = 0.037) and in newspapers/magazine (p-value < 0.001). The percentage of adolescents who reported having an item with a tobacco brand logo on it increased from 13.9% to 16.4%. The perception that boys and girls who smoked had more friends increased between 2000 and 2006 (p-values = 0.016 and 0.004 respectively). Current smoking was associated with male gender (OR = 1.55; 95% CI [1.09–2.19]), having smoking parents (OR = 1.75; 95% CI [1.23–2.50]), and smoking friends (OR = 14.94; 95% CI [8.61–25.92] for most or all friends smokers and OR = 4.38; 95% CI [2.93–6.56] for some friends smokers)).

**Conclusion:**

Results from this study indicate smoking was positively associated with male gender, having smoking friends or parents. We observed a slightly non significant increase in the prevalence of smoking between 2000 and 2006 among adolescents in Jamaica. Although there was a decrease in the rates of adolescents exposed to advertisement, the percentage of those who had an item with a tobacco brand logo had increased. The possible impact of the Jamaica's ratification of the Framework Convention on Tobacco control remains to be observed.

## Background

Tobacco use is a leading cause of preventable morbidity and mortality from non-communicable diseases [1-3]. Adolescent smoking is of public health significance as many adult smokers initiated the smoking habit as adolescents. Adolescent smoking is also important as it is associated with short-term health effects such as incident and exacerbation of asthma [4]. Smoking in adolescents may also be a marker of other unhealthy lifestyles or social problems such as alcohol use, illicit drug use, sedentary lifestyle, unprotected sex and truancy [5-9].

Globally, studies to estimate the prevalence of adolescent smoking and associated factors have been spurred by the Global Youth Tobacco Survey (GYTS) [10,11]. The GYTS was established by the World Health Organization (WHO) and Centers for Disease Control and Prevention's (CDC's) Office on Smoking and Health to track tobacco use among young people across countries using a standard methodology and core questionnaire. The aim of the GYTS surveillance system is to enhance the capacity of countries to design, implement, and evaluate tobacco control and public health prevention programs. Many of the published reports on Global Youth Tobacco Survey have been single year prevalence reports. However, as repeat surveys are conducted within the same settings (countries), there is opportunity to track the changes in prevalence of smoking and associated factors. In the case of Jamaica, the first round of the GYTS was conducted in 2000 and a repeat survey done in 2006. We therefore have two time points when data on adolescent smoking were obtained using nationally representative survey methods and common methodology. We therefore carried this study to estimate the prevalence of current cigarette smoking among in-school adolescents in Jamaica and associated factors using GYTS methodology in 2000 and 2006. Furthermore, we also conducted regression analysis to estimate the size of effect of association between current cigarette smoking and a list of explanatory variables. Data from the Jamaica GYTS were selected because this country has had a repeat GYTS, with the second survey having conducted just a few years following ratification of the Framework Convention on Tobacco Control (FCTC).

## Methods

We conducted secondary analysis of the Jamaican Global Youth Tobacco Survey (GYTS) implemented in 2000 and 2006. A full description of the data collection methods in the GYTS has been reported elsewhere [11]. Current cigarette smoking was defined as having smoked a cigarette, even a single puff, within the last 30 days preceding the survey. We were also interested to explore if the differences in the prevalence of smoking between the 2000 and 2006 survey. Data were analysed using SUDAAN software version 9 (Research Triangle Institute, Research Triangle Park, North Carolina, United States of America). Chi-square tests were used to compare differences between proportions.

## Results

1752 adolescents, 48.8% male and 51.2% females participated in the 2000 survey. In 2006, 1854 participated of whom 49.5 were males and 50.5% females. Table 1 indicates the prevalence of smoking among Jamaican school-going adolescents went up from 15.2% in 2000 to 16.7% in 2006, but this was not statistically significant (p = 0.22). The perception that smoking is not harmful increased 5% (from 10.9% to 15.9%). Reported parental smoking decreased from 39.4% to 35.5% in the same period of time.

**Table 1:** Prevalence of smoking among Jamaican adolescents in 2000 and 2006

	Year 2000	Year 2006	
Characteristic	% (95% CI)	% (95% CI)	P value

Age in years			0.92
Total	15.2 (13.4–17.3)	16.7 (12.9–18.6)	
=<13	11.6 (9.8–14.3)	15.5 (12.9–18.6)	
14	15.8 (9.2–25.7)	15.2 (12.2–18.7)	
15	20.9 (16.9–25.3)	18.2 (14.6–22.5)	
> = 16	22.9 (18.5–26.5)	21.7 (16.3–28.3)	
Gender (sex)			0.859
Females	11.7 (9.8–13.9)	12.0 (10.0–14.3)	
Males	19.3 (16.1–23.0)	21.8 (18.9–25.0)	
Parental smoking status			0.782
None	60.8 (57.8–64.5)	64.4 (61.8–66.4)	
One or both parents smokers	27.0 (22.7–31.0)	35.5 (33.1–38.4)	
Perception that smoking is harmful			0.890
Yes	10.9 (7.9–12.7)	15.9 (13.1–18.5)	
No	89.1 (86.5–91.7)	84.1 (81.9–86.3)	

Table 2 compares the rates of exposure to adverts between 2000 and 2006. There was a significant decrease in the prevalence of adolescents exposed to tobacco adverts on billboards (p-value = 0.037) and in newspapers/magazine (p-value < 0.001). The percentage of adolescents who reported having an item with a tobacco brand logo on it increased from 13.9% to 16.4%.

**Table 2:** Exposure to tobacco advertisements among Jamaican adolescents in 2000 and 2006

	Year 2000	Year 2006	
Characteristic			P value

Seen cigarette brand name on TV in past 30 days	76.9 (74.6–79.0)	67.4 (65.0–69.7)	0.080
Males	76.4 (72.6–79.7)	66.0 (62.5–69.3)	
Females	77.4 (74.6–80.0)	68.7 (65.5–71.8)	
Has item with cigarette brand logo	13.9 (12.2–15.8)	16.4 (14.6–18.3)	0.296
Males	15.7 (12.8–19.0)	19.4 (16.6–22.4)	
Females	12.2 (10.3–14.4)	13.5 (11.4–15.9)	
Seen tobacco adverts on billboards in past 30 days	66.0 (66.5–68.4)	62.9 (60.5–65.2)	0.037
Males	64.9 (60.8–68.7)	63.0 (59.5–66.3)	
Females	67.0 (64.0–69.9)	62.8 (59.5–66.0)	
Seen tobacco adverts in newspapers/magazines in past 30 days	61.4 (58.8–63.8)	56.5 (54.1–58.9)	<0.001
Males	61.7 (57.6–65.6)	56.2 (52.7–59.8)	
Females	61.1 (58.0–64.1)	56.8 (53.4–60.1)	

Table 3 indicates that the perception that boys and girls who smoked had more friends increased significantly between 2000 and 2006 (p-values = 0.016 and 0.004 respectively). There was no significant difference in the perception that boys or girls who smoked were attractive.

**Table 3:** Attitudes towards tobacco smoking distributed among Jamaican adolescents in 2000 and 2006

	Year 2000	Year 2006	
Characteristic	% (n)	% (n)	P value

Felt that boys who smoke have more friends	49.4 (45.4–51.5)	69.6 (66.7–72.3)	0.016
Males	44.8 (39.5–48.0)	64.4 (60.0–68.6)	
Females	54.3 (48.6–57.1)	75.0 (71.2–78.5)	
Felt like girls who smoke had more friends	25.2 (22.6–27.9)	35.9 (33.2–38.8)	0.004
Males	24.9 (21.0–29.3)	34.9 (30.9–39.0)	
Females	25.4 (22.3–29.0)	37.1 (33.2–41.2)	
Felt that boys who smoke are attractive	10.6 (8.9–12.6)	14.8 (13.0–16.9)	0.175
Males	13.8 (10.8–17.5)	17.9 (14.9–21.4)	
Females	7.8 (6.2–9.9)	12.2 (10.0–14.7)	
Felt that girls who smoke are attractive	8.9 (7.4–10.7)	11.1 (9.5–12.9)	0.327
Males	11.7 (9.1–15.1)	13.6 (11.0–16.4)	
Females	6.3 (4.8–8.2)	8.9 (7.0–11.2)	

Table 4 presents results from bivariate and multivariate analyses (2006). In bivariate analysis, current smoking was positively associated with male gender (OR = 2.04; 95% CI [1.56–2.68]), having smoking parents (OR = 2.38; 95% CI [1.80–3.16]), and smoking friends (OR = 21.99; 95% CI [13.99–34.57] for most or all friends smokers and OR = 5.38; 95% CI [3.80–7.61] for some friends smokers). The perception that smoking is harmful was associated with a non-significant decrease in the odds of smoking (OR = 0.83; 95% [0.57–1.23]). Results from the multivariate analysis were weaker than in bivariate analysis but with the same inference.

**Table 4:** Factors associated with current smoking among Jamaican adolescents in 2006

Characteristics	Unadjusted odds ratios [95% CI]	Adjusted odds ratios [95% CI]
Age (years)		
=<13	1.00	1.00
14	0.97 [0.69–1.37]	0.79 [0.50–1.25]
15	1.21 [0.86–1.71]	1.08 [0.68–1.73]
> = 16	1.51 [1.00–2.29]	0.92 [0.53–1.62]
Gender		
Female	1.00	1.00
Male	2.04 [1.56–2.68]	1.55 [1.09–2.19]
Parental smoking status		
None	1.00	1.00
One or both parents smokers	2.38 [1.80–3.16]	1.75 [1.23–2.50]
Best friend smokers		
None	1.00	1.00
Some	5.38 [3.80–7.61]	4.38 [2.93–6.56]
Most or all	21.99 [13.99–34.57]	14.94 [8.61–25.92]
Perception that smoking is harmful		
No	1.00	1.00
Yes	0.83 [0.57–1.23]	0.95 [0.57–1.58]

## Discussion

The prevalence of current cigarette smoking among in-school adolescents in 2000 and 2006 were 15.2% and 16.7% respectively (p = 0.22) i.e. the change was not statistically significant. The male and females GYTS prevalence estimates of current cigarette smoking among in-school adolescent were: 7.6% and 6.4% in Barbados (2002); 6.2% and 3.7% in Bahamas (2004); 18.9% and 10.4% in Belize (2002); 14.1% and 13.8% in Haiti (2005); 11.5% and 7.9% in St Lucia (2000) and 16.0% and 7.6% in Trinidad and Tobago (2000) [12]. Furthermore the prevalence of current cigarette smoking in-school adolescents had been reported at 1%–4.5% in Ethiopia, [10], 3.0% in Blantyre-Malawi [13], and 17.5% overall prevalence in the Americas [14].

We also observed that the proportion of adolescents who reported having a parent who was a smoker reduced, so did adolescents who reported having been exposed to pro-tobacco advertisements through billboards and magazines. However the proportion of adolescents who reported owning an item with a tobacco brand logo increased. It is difficult to determine the reasons for increases in some of the characteristics while there are increased in other pro-tobacco measures.

One observation regarding the Jamaican tobacco landscape is that the country signed into the World Health Organization's Framework Convention on Tobacco Control (FCTC) on 24 September 2003 and adoption followed on 7 July 2005. Currently, Jamaica has banned tobacco advertisements on local television and radio programs but advertisements from international programs have not been banned. There are also tobacco cessation programs at some health facilities with nicotine replacement therapy available. However, more still needs to be done as advertisements in local newspapers, magazines, billboards, tobacco brand logos on items not related to tobacco, public environmental tobacco smoking have not been banned. There are however, national tobacco control objectives and a dedicated agency for the control of tobacco [12].

We also found that current smoking among the adolescents was associated with having closest friends who were smokers, parental smoking and male gender. Perception that smoking was harmful to health was associated with reduced likelihood of smoking.

Previous studies on adolescent smoking conducted elsewhere have also reported positive relationship between peer smoking, parental smoking, male gender and smoking [10]. Parental smoking may influence adolescent smoking by facilitating the ready availability of cigarettes in the home and moderating parental attitudes towards adolescent smoking. Peer smoking could influence adolescent attitudes and smoking in a similar way as has been described for parental smoking. It is also possible that adolescents who are smokers may be more likely to choose other smokers as closest friends. However due to the cross sectional nature of data collection over the two surveys, it is not possible to assign any of the factors as causes of adolescent smoking. Furthermore, for non-time varying factors such as sex, we cannot really say that male sex is the cause of smoking but rather that being male may be associated with other intermediate factors that may explain male predominance of smoking.

We found that adolescents who perceived that smoking was harmful were less likely to smoke and those who perceived that smoking made youth attractive were less likely to smoke. According to the PRECEED/PROCEED model of health promotion individuals are likely to adopt a healthy behavior if they have the appropriate knowledge and attitude, are exposed to reinforcing and enabling factors such as friends, family and health workers [15]. We therefore suggest that intervention programs should aim to change adolescent behavior by highlighting the harmful effects of smoking. There is need to ensure that adolescents are not exposed to images that glamorize smoking. Negative attitudes towards smoking may influence eventual avoidance of smoking among adolescents.

It is however interesting to note that exposure to pro-tobacco advertisement on television, magazines and newspapers and billboards declined from 2000 to 2006. However, ownership of item with tobacco brand logo increased over the period under review. Whether the decline in prevalence of exposure to advertisement was related to bans on local television programs remain to be seen. Paradoxically, the proportion of adolescents with favorable perception towards smoking increased.

In a study of high school forms 4 and 5 and Jamaica, Siyobo and Lee [16] reported prevalence of cigarette smoking at 16.6%. Males, urban residents and adolescents whose parents were skilled professionals were more likely to smoke than rural, female and children of less skilled parents. A comparison of the Soyibo and Lee report [16] and our findings from the current study (years 2000 and 2006 surveys), suggest stability of the prevalence of cigarette smoking among adolescents in Jamaica.

## Limitations of the study

The limitations of the GYTS have been reported previously elsewhere [10]. Firstly data from the Global Youth Tobacco Survey are obtained through self-reports. As is the case with essentially all study methods where study participants self-report, there may be under-reporting as well as over-reporting. The GYTS aims to minimize such misreporting for emphasizing the collection of data through anonymous questionnaires. The possibility of mis-reporting cannot be entirely removed just by emphasizing anonymity although it can be reduced substantially.

Data from the GYTS are obtained only from students who are available in school that the survey is administered in the particular school. Students who are absent from school due to any reason are not followed up. Slonim-Nevo and Mukuka have reported that out of school adolescent were more likely to engage in unhealthy behaviours compared to in-school peers [17]. If this were also the case in Jamaica, then our results would be under-estimates. We however do not know whether this was the case or not; although it is important for the reader to keep this possibility in mind.

As the GYTS only recruits students that are enrolled in school, the findings may not be applicable to all adolescents in Jamaica as out of school adolescents may have prevalence estimates substantially different from adolescents who were enrolled in school. However, primary and secondary school enrolment ratios in Jamaica are 90% and 88% with 97% and 92% attendance ratios [18]. Any biases that may result because of non-enrolment and non-attendance at school are likely to be minimal. Our results can be described as fairly representative of the adolescent population in Jamaica.

Traditionally, trend analysis requires at least assessment of the prevalence at three different time points. Our study is based in two time points. However, even with the slight increase, a third reading that will show that trend is up may be more meaningful, a downward future prevalence will suggest inconclusive findings. We await future GYTS or similarly designed studies to assess whether there is a trend in prevalence of cigarette smoking among in school adolescents in Jamaica. In these future studies, it should be possible to use ARIMA (autoregressive integrated moving average) analysis to identify real changes having separated out noise from the results [19].

## Conclusion

We have demonstrated that the prevalence of current cigarette smoking among in school adolescents in Jamaica has risen slightly but not statistically significant. In fact the prevalence is relatively similar to previous estimate in the late 1990's. Predictors of smoking such as male gender and smoking status of parents or friends have either decreased on remained stable over the years. The recent signing in of Jamaica to the WHO's Framework Convention on Tobacco Control may provide the socio-political environment for significant reduction of tobacco use among adolescents in Jamaica.

## Competing interests

The authors declare that they have no competing interests.

## Authors' contributions

ER conducted the analysis, participated in the analysis of the data and drafting of the manuscript.

ASM conceived the data analysis plan and participated in the interpretation of the findings and drafting of the manuscript.

SS participated in the interpretation of the findings and drafting of manuscript.

All authors read and approved the final draft of the manuscript.
